# Donor site morbidity after computer assisted surgical reconstruction of the mandible using deep circumflex iliac artery grafts: a cross sectional study

**DOI:** 10.1186/s12893-022-01899-z

**Published:** 2023-01-09

**Authors:** Leonard Simon Brandenburg, Pit Jacob Voss, Thomas Mischkowsky, Jan Kühle, Michael Andreas Ermer, Julia Vera Weingart, René Marcel Rothweiler, Marc Christian Metzger, Rainer Schmelzeisen, Philipp Poxleitner

**Affiliations:** 1 Department of Oral and Maxillofacial Surgery, Clinic, Medical Center-University of Freiburg, Faculty of Medicine, University of Freiburg, Hugstetterstr. 55, 79106 Freiburg, Germany; 2 Department of Orthopedics and Trauma Surgery, Clinic, Medical Center-University of Freiburg, Faculty of Medicine, University of Freiburg, Hugstetterstr. 55, 79106 Freiburg, Germany

**Keywords:** Mandible reconstruction, Deep circumflex iliac artery, Comorbidity

## Abstract

**Background:**

Computer Assisted Design and Computer Assisted Manufacturing (CAD/CAM) have revolutionized oncologic surgery of the head and neck. A multitude of benefits of this technique has been described, but there are only few reports of donor site comorbidity following CAD/CAM surgery.

**Methods:**

This study investigated comorbidity of the hip following deep circumflex iliac artery (DCIA) graft raising using CAD/CAM techniques. A cross-sectional examination was performed to determine range of motion, muscle strength and nerve disturbances. Furthermore, correlations between graft volume and skin incision length with postoperative donor site morbidity were assessed using Spearman's rank correlation, linear regression and analysis of variance (ANOVA).

**Results:**

Fifteen patients with a mean graft volume of 21.2 ± 5.7 cm^3^ and a mean incision length of 228.0 ± 30.0 mm were included. Patients reported of noticeable physical limitations in daily life activities (12.3 ± 11.9 weeks) and athletic activities (38.4 ± 40.0 weeks in mean) following surgery. Graft volume significantly correlated with the duration of the use of walking aids (R = 0.57; p = 0.033) and impairment in daily life activities (R = 0.65; p = 0.012). The length of the scar of the donor-site showed a statistically significant association with postoperative iliohypogastric nerve deficits (F = 4.4, p = 0.037). Patients with anaesthaesia of a peripheral cutaneous nerve had a larger mean scar length (280 ± 30.0 mm) than subjects with hypaesthesia (245 ± 10.1 mm) or no complaints (216 ± 27.7 mm).

**Conclusions:**

Despite sophisticated planning options in modern CAD/CAM surgery, comorbidity of the donor site following  iliac graft harvesting is still a problem. This study is the first to investigate comorbidity after DCIA graft raising in a patient group treated exclusively with CAD/CAM techniques. The results indicate that a minimal invasive approach in terms of small graft volumes and small skin incisions could help to reduce postoperative symptomatology.

*Trial registration* Retrospectively registered at the German Clinical Trials Register (DRKS-ID: DRKS00029066); registration date: 23/05/2022

## Background

Oral squamous cell carcinoma (OSCC) is the sixth most common cause of death among all cancer-related diseases with an increasing incidence, [1] especially in younger patients [2]. In 2018, approximately 700,000 new cases and 350,000 deaths due to OSCC were estimated worldwide, making it a global health issue [3]. Especially advanced cases of OSCC need fast and radical treatment to enable adequate long-term survival [3–5].

In surgical treatment of OSCC, tumor-free resection is aspired and therefore radical surgery is performed [3, 6]. OSCC mostly affects the mandible, [7] therefore resections of the mandible are frequently required, leading to large defects of the lower jaw [8]. Subsequent plastic reconstruction of the mandible is crucial to enable a proper rehabilitation of the stomatognathic system including mastication, deglutition and speech as well as the aesthetic appearance of the face [9–11]. The current gold standard in reconstructing bony defects of the mandible are microvascular free flaps [3].

After the introduction of microsurgical techniques, different donor-sites were described for harvesting of osteocutaneous free flaps [12]. The first successful free flap surgery was performed using an autotransplant of omentum to a large scalp defect [13]. Hidalgo first described the free fibula flap (FFF) as a microvascular transplant to be used in the head and neck area [14]. The scapula osteocutaneous free flap (SOFF) and the iliac crest flap supplied by the deep circumflex artery (DCIA) present valuable alternatives for bony reconstruction in maxillofacial surgery and were described shortly after [15, 16]. Depending on the localization and the size of the defect, the choice of specific graft may provide particular advantages in the reconstruction process. The FFF has emerged to be the workhorse flap in the reconstruction of the mandible. Due to the wide section of dense cortical bone supplied by the fibula, it became indispensable when forming a neo-mandible [17]. The SOFF, on the other hand, is used preferably for the reconstruction of the temporomandibular joint, as the scapular tip can be used for anatomical replacement of the condylar head. Moreover, by raising the latissimus dorsi muscle, a high volume of soft tissue can be harvested alongside the SOFF [18, 19]. The DCIA graft provides anatomically shaped bone with sufficient vertical height resembling the anatomical form of the mandibular body [20]. Therefore, it offers excellent conditions for subsequent placement of osseointegrating dental implants [21, 22]. Due to its favorable characteristics some authors even proposed the DCIA graft as transplant of choice for reconstruction of the mandible [10, 11].

Due to the widespread use of computed tomography (CT) and computer-assisted image processing, the options for preoperative planning have improved significantly in recent decades [23–25]. Computer aided design and computer aided manufacturing (CAD/CAM) were introduced to maxillofacial surgery by Hirsch et al. and continuously improved plastic reconstruction in head and neck surgery [26]. By segmentation of the anatomical structures and three-dimensional visualization of the surgical sites, meticulous preoperative planning can be performed using CAD/CAM. Besides mere visualization purposes CAD/CAM transfer the surgical plan into the operation theatre using 3D-printed drilling and cutting guides and can therefore facilitate surgery [27].

Besides considerations focusing on the oncological as well as the functional and aesthetic outcome of the stomatognathic system, comorbidities of the donor-site have to be taken into account. Current literature report controversial results regarding donor-site morbidity after harvesting of DCIA graft and is mostly based on retrospective reviews of medical records. Chronic pain, gait disturbances and neurosensory deficits are only a few of the known adverse effects described in the context of DCIA graft reconstructions [20, 28, 29]. However, to date only few studies investigate donor-site morbidity after CAD/CAM driven DCIA graft harvesting. Since CAD/CAM techniques have only been used in clinical routine for a decade and OSCC patients face limited long-term survival rates, data investigating the donor-site morbidity by physical follow-up is difficult to acquire [20].

The first objective of this study is to investigate the extent of donor site morbidity in a study group receiving CAD/CAM driven DCIA graft reconstruction of the mandible to allow comparison with existing publications reporting donor site morbidity in conventional cases. The second aim of this study is to assess if there is a correlation of the graft volume or the incision length with postoperative donor-site morbidity following CAD/CAM driven DCIA graft harvesting. For this purpose, thorough chart review, evaluation of imaging data and postoperative physical examination was performed.

## Materials and methods

This study was conceived in accordance with the Declaration of Helsinki and has been approved by the ethics committee of the Albert-Ludwigs-University Freiburg, Germany (573/19). All participants gave written informed consent for study implementation. The structuring of the manuscript was performed regarding to the STROBE guidelines [30].

### Study group

This cross-sectional study investigated donor-site morbidity of patients who received a CAD/CAM driven DCIA graft reconstruction of the mandible following resection of OSCC in our single tertiary center (Clinic of Oral and Maxillofacial Surgery, Medical Center— University of Freiburg, Germany). Patients who underwent CAD/CAM driven DCIA graft reconstruction of the mandible were identified by thorough review of all electronic patient charts of our clinic and were invited for physical follow-up examination. At the time of study conception, CAD/CAM was already introduced as standard operating procedure for bony reconstruction after ablation of OSCC at our clinic. Conventional operations were only performed in exceptional cases and could therefore not be investigated as a control group.

### Inclusion and exclusion criteria

Patients included in this study were at least 18 years old, signed a written declaration of consent, could attend an appointment for physical examination and were able to cooperate during physical examination. The period between surgical treatment and physical examination had to be at least twelve months.

Exclusion criteria were as follows: patients under 18 years of age, patients who did not agree with study implementation and patients with previous surgeries in the pelvic area. Reconstructions of the mandible due to diseases other than OSCC were excluded to rule out a confounding influence caused by different diagnoses. Secondary reconstructions, or reconstructions which were performed without the use of CAD/CAM techniques were excluded likewise.

### Preoperative planning procedure

**Fig. 1:**
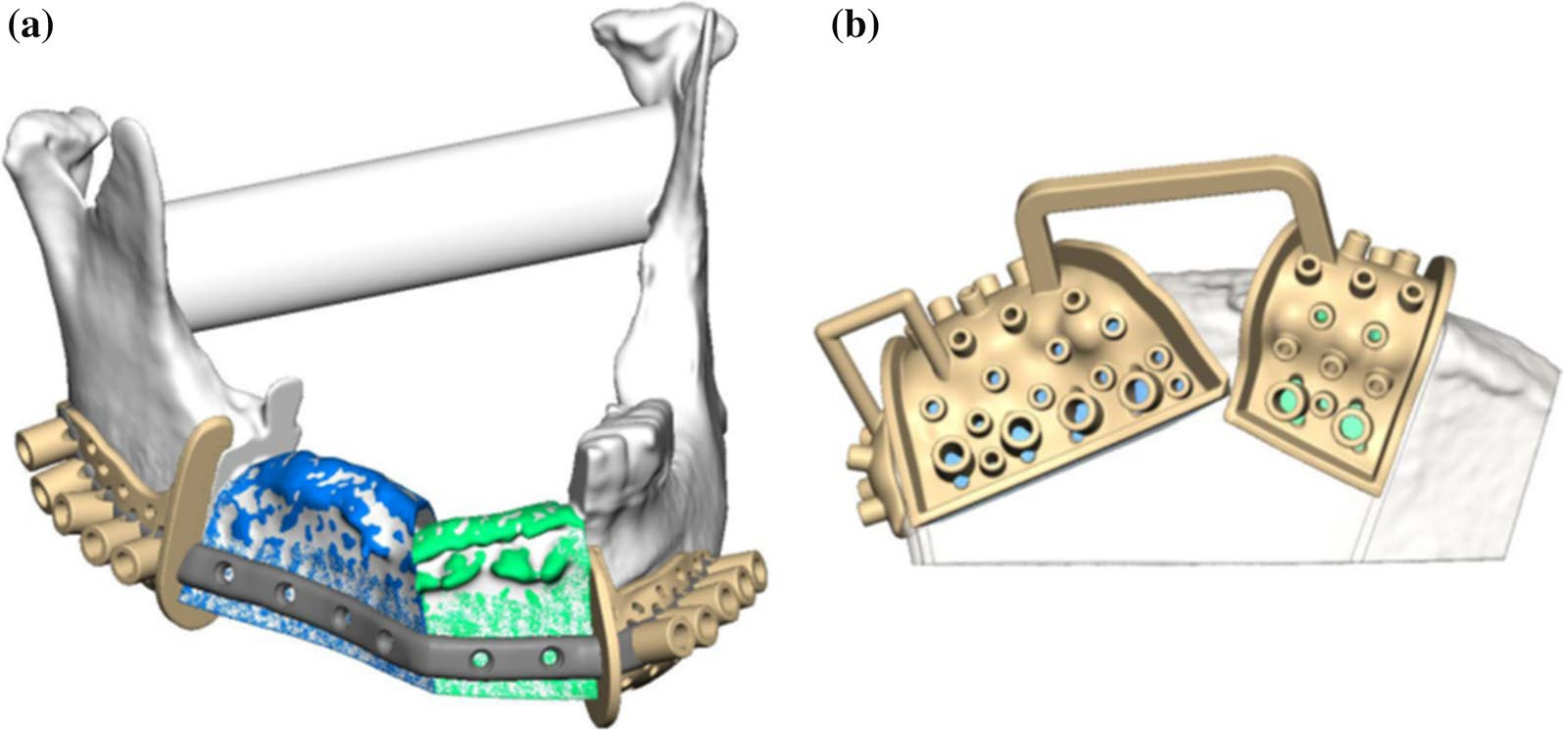
Preoperative planning of best possible mandibular reconstruction (**a**) by choosing the most fitting sections of the iliac crest (**b**) and subsequent design of cutting guides (beige) and a patient individual osteosynthesis plate (grey)

Preoperative planning was performed in collaboration with a technician of the virtual planning vendor (KLS Martin Group, Tuttlingen, Germany) during a web meeting using high-resolution CT scans of the head, neck and pelvis (slice thickness ≤ 1 mm). The underlying CT data was segmented and displayed for virtual 3D-visualization. Considering the patient-specific anatomy, the resection margins, the section and size of the iliac crest used for graft harvesting and, if necessary, osteotomies within the graft were determined (Fig. 1). Afterwards fitting of the planned graft with the resected mandible was virtually assessed. Once planning was complete, sawing and drilling guides and an individual osteosynthesis plate for surgical transfer of the virtual plan and fixation of the graft respectively were designed and manufactured.

### Surgical procedure and inpatient care

Surgery was performed under general anesthesia using a two-team approach to reduce surgery time. Team time-out was carried out before surgery. Graft harvesting was always performed by the same team.

### Raising of the microvascular DCIA graft

**Fig. 2:**
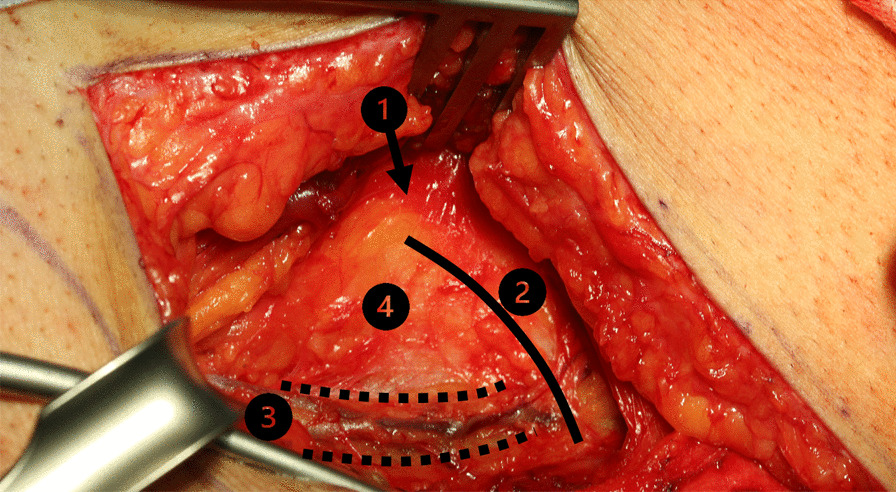
Exposure of the iliac crest after careful preparation of the overlying soft tissue layers. (1) Anterior superior iliac spine. (2) Iliac crest. (3) Branch of the deep circumflex iliac artery. (4) Pelvic bone

**Fig. 3:**
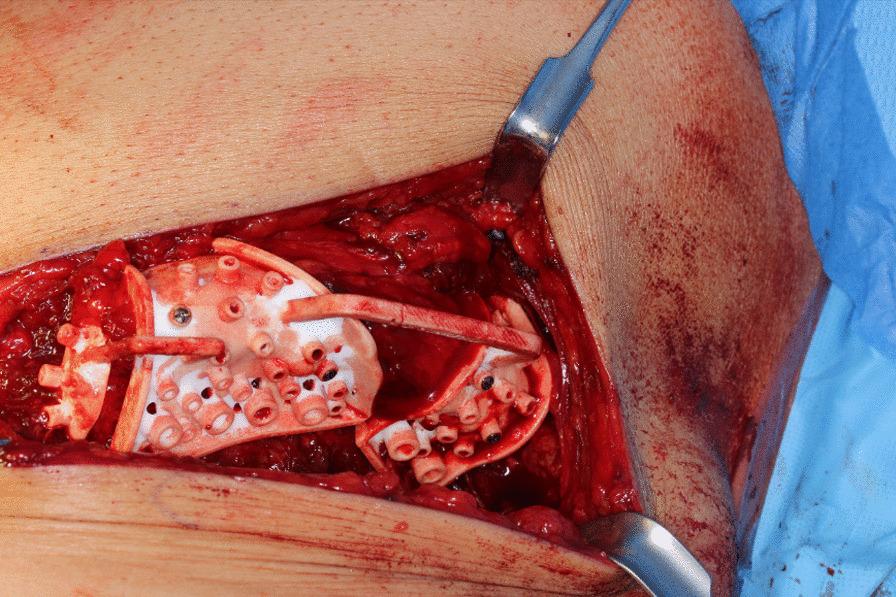
Insertion and fixation of the patient individual cutting and drilling jig. The correctly adapted cutting guide allows realizing the preoperative plan and acquiring the best possible configuration of the graft

**Fig. 4:**
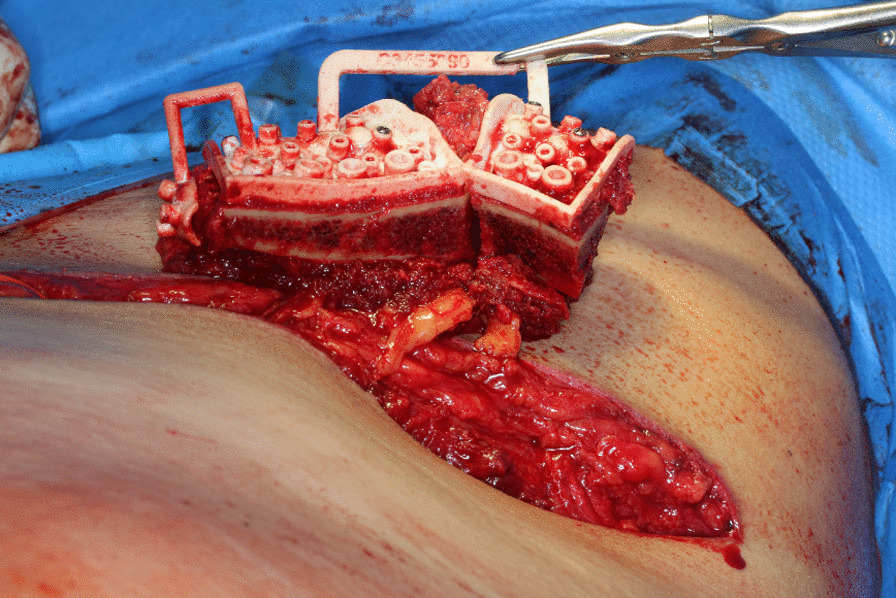
Iliac crest after osteotomy and withdrawal from the pelvis. The vessels are not cut until resection is completed to guarantee shortest possible ischemia time

**Fig. 5:**
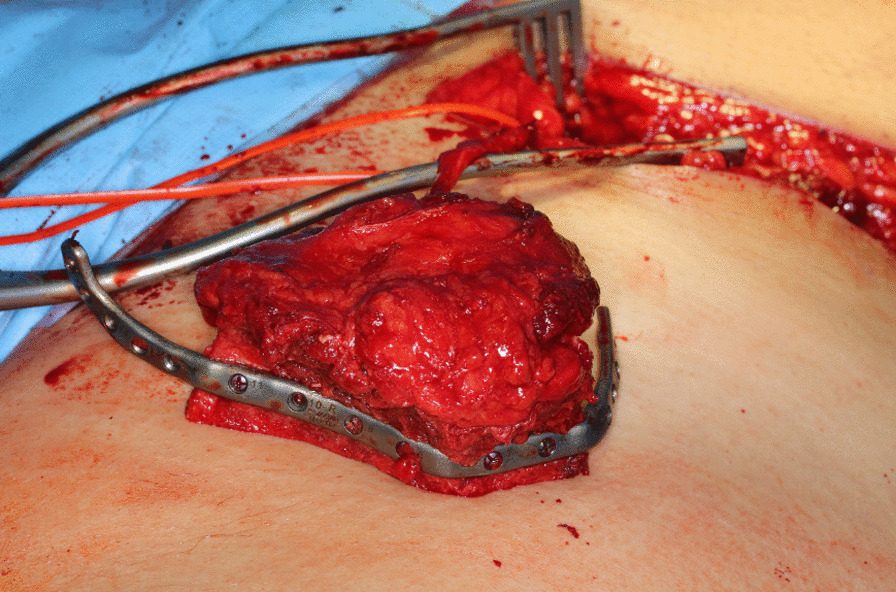
Iliac crest after completion of internal osteotomies and fixation of the miter-cut parts along the patient individual osteosynthesis plate. The pedicle is marked with a red vessel-loop

After standardized surgical site skin preparation and application of local anesthesia, a premarked skin incision was made. The abdominal fascia was exposed and incised one fingerbreadth above the inguinal ligament. The deep circumflex iliac artery was located and vessels to the iliac artery were traced. Transection of the externus, internus and transversus abdominis muscle was performed. Subsequently, the externus abdominis muscle, the gluteus medius muscle and the tensor fascia latae muscle were detached from the external side of the iliac crest using a raspatorium (Fig. 2). The 3D-printed cutting guide was placed in the planned anatomical position (Fig. 3). Osteotomies and predrilling of the screw-holes were performed as specified by the guide (Fig. 4). The patient specific osteosynthesis plate was fixated to the bone graft using the predrilled holes (Fig. 5). Afterwards, the iliacus muscle was detached from the iliac surface of the graft. The graft was stored in moist sterile cloths while the vessel remained pedicled, until the graft was needed at the resection site. Once the pedicle vessels were cut, wound closure was performed using sutures to fixate the iliacus, transversus and internus muscle to the iliac fossa first. The externus muscle and the abdominal fascia were sutured subsequently. The suture of the skin was completed with multiple layer sutures of the subcutis and skin.

### Inpatient care

Patients were transferred to intensive care unit (ICU) for airway monitoring after surgical treatment. The endotracheal tube was usually removed within the first 2 days after surgery if the swelling of the oral cavity allowed spontaneous breathing. As soon as the patient was stabilized hemodynamically and endotracheal tube was removed, transfer to surgical ward was conducted. In the first week after surgery, nutrition was managed using a nasogastric tube. If the patient was not able to eat soft or liquid food for longer than 1 week, percutaneous endoscopic gastrostomy was performed. During stay in surgical ward, speech and language therapists as well as physical therapists performed daily therapy to enable fast rehabilitation. Postoperative pain was managed using non-steroidal anti-inflammatory drugs and transdermal fentanyl patches (usually 12 µg/h every third day).

### Data acquisition

After review of all documented surgeries performed at the Clinic of Oral and Maxillofacial Surgery, Medical Center— University of Freiburg, Germany in a period between June 2017 and June 2020, patients who underwent CAD/CAM driven DCIA graft reconstruction of the mandible were identified. All collected data was saved in a spreadsheet (Microsoft Excel^®^ Version 16.0, Microsoft Corporation, Albuquerque, NM, USA).

### Review of patient charts and imaging data

By reviewing all electronic patient charts, information regarding epidemiologic data (sex, age at the time of surgery), hospital stay (length of stay at ICU and surgical ward), surgical therapy (operating time, ischemic time, blood loss and weight of resected tumor sample) and postoperative outcome parameters was collected. Postoperative imaging data was used to determine the volume of the raised DCIA graft. Using the open source software 3D slicer the CT-scan was segmented and the volume of the inserted graft was determined by volumetric measurement of the graft [31].

### Interviews

Before physical examination a structured interview was conducted by one investigator (TM) using a predefined questionnaire. The questionnaire included questions regarding general health conditions (nicotine or alcohol consumption, autoimmune diseases, diabetes mellitus) and postoperative features of daily life (postoperative pain, intake of analgesics, limitation in daily or physical activities and use of walking aids).

### Physical examination

Physical examination was performed by one investigator (TM) on both legs in order to directly compare the operated leg with the unaffected leg. (1) The range of motion (ROM) of the hip joint was measured using a goniometer and given as angular degrees for the following directions of movement: flexion/extension, abduction/adduction, and interior rotation/external rotation. (2) The muscle strength during hip flexion was determined using the muscle function test according to Janda [32]. (3) The sensitivity of peripheral cutaneous nerves of the leg and pelvic area (iliohypogastric, ilioinguinal, genitofemoral and lateral femoral cutaneous nerve) was performed using a cotton bud. Moreover, the length of the scar at the donor-site was determined with a ruler, as it corresponds to the length of the surgical skin incision.

### Statistical analysis

The collected data was transferred into the Statistical Package for the Social Sciences ^®^ Version 26.0 for further analysis (IBM SPSS ^®^ Statistics for Windows, Version 26.0. Armonk, NY, IBM Corporation). Variables were tested for normal distribution using the Shapiro–Wilk test and for homoscedasticity using Levene’s test where appropriate. Associations between variables scaled ordinal were tested for statistical significance using Spearman’s rank correlation. The influence of metric-scaled predictors on equally metric-scaled clinical end points was also examined in the form of multiple linear regression. Group comparisons were performed determining an independent categorical variable and a dependent metric-scaled variable using single-factor analysis of variance (ANOVA). The influence of age, sex, graft volume, graft diameter, and length of skin incision on donor-site-morbidity was examined using Spearman’s rank correlation, multiple linear regression, or analysis of variance (ANOVA). In contrast, for connected samples, the nonparametric Wilcoxon signed-rank test was used. The significance level was set to p = 0.05 in all cases.

## Results

### Study group characteristics

After an initial review of all documented surgeries during the mentioned period, 38 patients were eligible for study implementation. Six patients were not available, two patients could not travel to our clinic, 13 patients refused to participate in the study, and two patients died before their examination date. Eventually fifteen patients (five women and ten men) with a mean age of 65.5 ± 10.3 years (minimum: 53; maximum: 84 years) could be included in this study.

### Review of patient charts and imaging data

Operating time ranged from 5 to 16 h, with a mean of 11.8 ± 3.0 h. Ischemic time was between 60 and 160 min with a mean of 93.5 min. Blood loss was between 300 and 2500 ml with a mean of 1021 ml ± 582 ml. Patients stayed at the ICU and the surgical ward for 5.7 ± 3.5 days and 25.8 ± 15.0 days respectively. The volume of the raised DCIA graft ranged between 12.1 cm^3^ and 30.3 cm^3^ with a mean of 21.2 ± 5.6 cm^3^. Raising of a soft tissue bulk alongside the DCIA graft was performed in one-third (5/15) of all patients. The weight of the resected tumor samples was specified in the histopathological report and was between 17.5 g and 98 g with a mean of 65 g ± 31 g. Harvesting of the anterior superior iliac spine (ASIS) was performed in 12 out of 15 cases. Postoperative development of hernia was detected in three patients. No statistically significant difference regarding the ROM, muscle strength or occurrence of neurosensory deficits were found between patients who did develop postoperative hernia and those who did not. Local infection of the donor site was found in three patients. Two patients required revision of surgery during the follow-up period. No complication due to local hematoma of the transplant site wad documented. Meralgia paresthetica and paralytic ileus was not documented in any cases. Local recurrence occurred in one patient. One patient died during the follow-up period due to cardiovascular disease.

### Interviews

Five patients confirmed regular alcohol consumption, seven patients reported of regular smoking. One patient suffered from diabetes mellitus, two patients of autoimmune diseases. Patients reported of physical limitations in postoperative daily life activities for 12.3 ± 11.9 weeks in mean. Athletic activities were impossible for 6–15 weeks after surgery (38.4 ± 40.0 weeks in mean). Walking aids were used for 2–115 weeks postoperatively (37.1 ± 44.8 weeks in mean). Analgesics were required for a mean of 10.1 ± 6.5 weeks. At the time of examination, patients mostly reported no or low pain sensation of the hip (visual analog scale mean 0.3 ± 0.8). Table 1 gives an overview of anamnestic features regarding general health conditions. Table 2 summarizes study group characteristics investigated by chart review.

**Table 1:** Anamnestic features regarding general health conditions reported by the patients during interview (SD = standard deviation)

Parameter	Yes	No
Alcohol consumption	5 (33.3%)	10 (66.7%)
Smoking	7 (46.7%)	8 (53.3%)
Diabetes mellitus	1 (6.7%)	14 (93.3%)
Autoimmune disease	2 (13.3%)	13 (86.7%)

**Table 2:** Characteristics of the study group regarding therapy and postoperative rehabilitation identified by interview, chart review and evaluation of postoperative CT-scans (SD = standard deviation)

Parameter	Mean ± SD	Minimum	Maximum
Length of skin incision in mm	228 ± 29.93	180	280
Graftat volume in cm^3^	21.2 ± 5.6	12,1	30.3
Duration of surgery in hours	11.8 ± 3.00	5	16
Duration of in-hospital stay in days	25.80 ± 15.02	4	50
Duration of stay at ICU in days	5.67 ± 3.50	1	11
Impairment in daily life activities in weeks	12.27 ± 11.87	3	52
Impairment in athletic activities in weeks	38.40 ± 39.66	6	115
Duration of the use of walking aids in weeks	37.07 ± 44.76	2	115
Duration of intake of painkillers in weeks	10.14 ± 6.46	4	26
Visual analogue scale at day of examination (no unit specified)	0.33 ± 0.82	0	3

### Physical examination

Range of motion (ROM) and muscle strength of the hip joint, as well as neurosensory deficits of the unaffected and operated side were examined and are presented in Tables 3, 4, 5. The length of the scar at the graft site amounted 228.0 ± 30.0 mm in mean (minimum: 180 mm; maximum: 280 mm).

**Table 3:** Differences between the surgical and unaffected side regarding ROM of the hip joint given in degree of angle (SD = standard deviation)

Parameter	Mean ± SD	Minimum	Maximum
Extension	0.00 ± 1.89	− 5	5
Flexion	− 10.00 ± 14.88	− 50	10
Abduction	0.00 ± 11.18	− 20	5
Adduction	− 0.33 ± 4.42	− 10	5
Internal rotation	− 5.00 ± 5.67	− 15	0
External rotation	0.67 ± 8.84	− 10	20

**Table 4:** Comparison of the muscle strength of the surgical and unaffected side during flexion of the hip joint given by the total number and proportion of patients (in brackets) with no differences between both sides, weaknesses at the surgical side and weaknesses at the unaffected side (SD = standard deviation)

Motion	No differences	Weakness at surgical side	Weakness at unaffected side
Extension	13 (86.7%)	2 (13.3%)	0 (0%)
Flexion	6 (40%)	9 (60%)	0 (0%)
Abduction	9 (60%)	6 (40%)	0 (0%)
Adduction	14 (93.3%)	1 (6.7%)	0 (0%)
Internal rotation	11 (73.3%)	4 (26.7%)	0 (0%)
External rotation	9 (60%)	6 (40%)	0 (0%)

**Table 5:** Comparison of the sensibilitiy of cutaneous peripheral nerves of the surgical and unaffected side given by the total number and proportion of patients (in brackets) with no differences between both sides, hypesthesia at the surgical side and anesthesia at the surgical side (SD = standard deviation)

Peripheral cutaneous nerve	Physiologic sensibility	Hypesthesia	Anesthesia
*Lateral cutaneous nerve of thigh*	4 (26.7%)	8 (53,3%)	3 (20%)
*Genitofemoral nerve*	10 (66.7%)	5 (33,3%)	0 (0%)
*Iliohypogastric nerve*	10 (66.7%)	4 (26,6%)	1 (6,7%)
*Ilioinguinal nerve*	8 (53.3%)	7 (46,7%)	0 (0%)

### Statistical analyses

#### Parameters acquired by interview

Graft volume significantly correlated with the duration of the use of walking aids (R = 0.57; p = 0.033) and impairment in daily life activities (R = 0.65; p = 0.012). There was no statistical correlation of sex, substance abuse, medical preconditions, age, incision length or transplant volume with duration of hospital stay, pain sensation or intake of analgesics.

#### Range of motion

Comparing the ROM of the hip joint of the operated leg with the unaffected leg, the Wilcoxon signed-rank test showed significant lower values in flexion (− 10.0° ± 14.9°; *p* = 0.012) and internal rotation (− 5.0° ± 5.7; *p* = 0.006) of the operated leg. There were hardly any differences when examining abduction, adduction, external rotation, and extension (Table 6). The period between surgery and physical examination did not significantly correlate with differences in ROM of the hip joint between the operated and the unaffected leg. No significant difference was found between men and women in the range of motion of the hip joint of the operated leg or its difference from the unaffected side. Alcohol consumption (p < 0.05) and smoking (p < 0.01) were significantly correlated with the occurrence of a smaller ROM during abduction. Age correlated significantly with differences in extension (R = − 0.53; p = 0.042) and abduction (R = 0.656; p = 0.008) between the operated and the unaffected leg. Graft volume was correlated significantly with differences in internal rotation (R = − 0.594; p = 0.025) between the operated and the unaffected leg.

**Table 6:** Spearman’s rank correlation coefficients for patient specific factors age (in years), follow-up interval (in weeks), length of skin incision (in cm), graft diameter (in cm), graft volume (in cm^3^) and difference of ROM of the hip joint between the operated and the unaffected side

Difference of ROM	Age	Follow-up	Skin incision	Graft diameter	Graft volume
In extension (°)	**− 0.530 **	− 0.064	− 0.042	− 0.255	0.094
In flexion (°)	0.086	− 0.193	0.095	− 0.135	0.011
In abduction (°)	**0.656**	− 0.025	0.32	0.104	0.007
In adduction (°)	0.344	− 0.331	− 0.244	0.048	0.265
In internal rotation (°)	0.219	0.092	− 0.167	0.162	**− 0.594 **
In external rotation (°)	0.169	0.255	0.378	0.373	− 0.408

Significance level of the correlation is given by the according *p*-value. *p*-values ≤ 0.05 are written bold

#### Muscle strength

In the investigation of muscle strength during flexion of the hip joint according to Janda, the operated leg revealed to be less powerful in all directions of movement, especially during flexion. Women (5/5) were afflicted more frequently by a weakness of the operated leg than men (6/10; p < 0.05). Moreover, alcohol consumption (p < 0.05) and smoking (p < 0.01) correlated significantly with a detectable weakness of the operated leg. No statistical correlations could be found between age, graft volume, length of skin incision and muscle strength (Table 7).

**Table 7:** Evaluation of the differences of muscle strength during flexion of the hip joint between the operated and unaffected side in dependence of patient specific factors age (in years), follow-up interval (in weeks), length of skin incision (in cm), graft diameter (in cm and, graft volume (in cm^3^) using ANOVA

Difference of muscle strength	Age	Follow-up	Skin incision	Graft diameter	Graft volume
In extension	0.165	0.944	0.53	0.724	0.309
In flexion	0.302	0.333	0.334	0.445	0.092
In abduction	0.689	0.278	0.125	0.795	0.248
In adduction	0.217	0.962	0.098	0.718	0.668
In internal rotation	0.377	0.536	0.07	0.447	0.219
In external rotation	0.342	0.971	0.084	0.138	0.747

Significance level of the correlation is given by the according *p*-value. No significant correlation could be identified

#### Sensitivity of the peripheral cutaneous nerves

Testing the sensitivity of the peripheral cutaneous nerves, reduction in sensation (n = 8), as well as loss of sensation (n = 3) of the lateral cutaneus femoris nerve was noticeable (Table 8). The length of the scar of the donor-site showed a statistically significant association with postoperative iliohypogastric nerve deficits (F = 4.4, p = 0.037). Patients with anaesthaesia of a peripheral cutaneous nerve had a larger mean scar length (280 ± 30.0 mm) than subjects with hypaesthesia (245 ± 10.1 mm) or no complaints (216 ± 27.7 mm). There was no significant difference found between men and women regarding neurosensory deficits. In contrast, alcohol consumption (p < 0.01) and smoking (p < 0.01) correlated significantly with the occurrence of neurosensory deficits.

**Table 8:** Evaluation of the differences of nerve disturbances at the operated side in dependence of patient specific factors age (in years), follow-up interval (in weeks), length of skin incision (in cm), graft diameter (in cm) and graft volume (in cm^3^) using ANOVA

Disturbance of sensibility	Age	Follow-up	Skin incision	Graft diameter	Graft volume
Lateral cutaneous nerve of thigh	0.405	0.585	0.437	0.491	0.113
Genitofemoral nerve	0.246	0.191	0.542	0.691	0.777
Iliohypogastric nerve	0.259	0.842	**0.037**	0.28	0.405
Ilioinguinal nerve	0.099	0.369	0.553	0.486	0.119

Significance level of the correlation is given by the according *p*-value. *p*-values ≤ 0.05 are written bold

## Discussion

In this study the extent of donor site morbidity in patients receiving CAD/CAM driven reconstruction of the mandible using DCIA grafts was documented. A correlation between the volume of the raised DCIA graft and postoperative use of walking aids and impairment in daily life activities could be found. Moreover, the incision length correlated with the occurrence of neurosensory deficits of the iliohypogastric nerve. The severity of detected neurosensory deficits correlated with the length of the skin incision: anesthesia of peripheral cutaneous nerves was associated with long skin incisions (280 ± 30.0 mm), whereas patients with hypesthesia and normal neurosensory function had shorter skin incisions (245 ± 10.1 mm and 216 ± 27.7 mm respectively). These results confirm that surgery should be performed as minimally invasive as possible to reduce postoperative donor-site morbidity.

Liu et al. found a slightly smaller incision lengths of 186 mm in mean in a study group of CAD/CAM treated patients, [33] which is comparable to the incision length reported by Schardt et al. who investigated conventionally treated patients [34]. It must be questioned critically if conventional operation techniques represent the more minimal invasive approach. The use of CAD/CAM techniques may require larger surgical incisions to insert the cutting guide and the instruments needed for harvesting the pelvic bone. As neurosensory deficits are one of the major postoperative morbidities reported in literature, the prescription of vitamin B complex and similar medication could be considered to improve nerve regeneration [35].

ROM of the hip joint of both legs was investigated during physical examination to quantify the impairment induced by surgery. Flexion and internal rotation were decreased significantly (-10.0° ± 14.9° and − 5.0° ± 5.7 respectively) in comparison to the unaffected leg, while other directions of motion were hardly affected after graft harvesting (Table 2). This shows that raising of DCIA grafts has detrimental effects on mobility of the hip in general and predominantly affects specific motions. As harvesting of the anterior superior iliac spine (ASIS) was frequently performed to obtain an anatomical reconstruction of the mandible (12/15), contraction of muscles originating from the ASIS could cause pain after surgery. Therefore, a significant restriction of flexion and internal rotation may be explained by painful sensations at the ASIS caused by the sartorius and tensor fasciae latae muscle e.g.[36]. To forgo postoperative donor-site morbidity due to extensive resection of the iliac crest or the ASIS, a more minimally invasive workflow should be considered. Modabber et al. described a medial approach for harvesting a DCIA graft, which enables to preserve not only the ASIS, but also the iliac crest. Therefore the gluteus medius, the tensor fasciae latae, the sartorius muscles are left in place and do not become cut or stripped [37]. This may also have an impact on the development of postoperative hernia, because the iliac crest is an important anatomical structure for inserting trunk muscles, which are crucial for the stability of the anterior and lateral abdominal wall.

ANOVA was used to investigate further relations of ROM with patient-specific factors (Table 6). Controversially older patients experienced pronounced impairment in abduction of the operated leg (R = 0.656; p = 0.008), while age and graft volume correlated with smaller deficits in extension (R = − 0.53; p = 0.042) and internal rotation, (R = − 0.594; p = 0.025). Even though a recent retrospective study reported no significant correlation between age and donor-site morbidity, [38] general physical impairment of the elderly may reasonably explain a more severe impairment after surgery. Nonetheless, there is no reasonable explanation why older patients, or patients who underwent more extensive DCIA graft harvesting should experience fewer postoperative restriction of ROM. The most likely reason for these contentious results may be the weak validity of goniometric measurements and the small sample size of this study [39]. Pre- and postoperative CT- or MR-data of the donor sites could be used to more precisely and objectively reflect the comorbidity after DCIA graft harvest. This method is already frequently used in sports medicine to enable functional and anatomical comparisons and should be considered in future studies [40].

Investigation of muscle strength equally showed that the performed surgery using CAD/CAM impairs functionality on the operated leg. Especially flexion (60% of patients), abduction (40% of patients) and internal rotation (27% of patients) were weaker in comparison to the opposite leg (Table 7). Likewise, low significance of the results may be explained by the low validity of the applied examination technique and its susceptibility depending on the individual perception of the rater [41, 42]. There was no significant impact of graft volume on measurements regarding ROM and perceived muscle strength of the leg.

In the last 2 years, three systematic reviews were published summarizing the findings regarding donor-site morbidity after head and neck reconstruction of the last 20 years [20, 28, 29]. Despite the abundance of existent publications, [21, 33, 34, 38, 43–53] only few studies are available, which investigate the impact of graft volume on donor-site morbidity after mandibular reconstruction using DCIA grafts [38, 54, 55]. None of them performed physical examinations of patients. In comparison to the average raised bone volume described in literature (15 cm^3^), this study presents a study group with relatively high mean graft volume of 21.17 ± 5.65 cm^3^ [55].

Katz et al. performed a retrospective chart review of DCIA graft patients and could acquire information about the graft volume in 65% of the investigated cases. A mean graft volume of 8.4 ± 6.9 cm^3^ was found [38]. No specifications concerning the applied surgical technique (CAD/CAM vs. conventional surgery) was made. The volume of the bone graft as stated in the reviewed charts was significantly correlated with a longer hospital stay. No correlation of graft volume with functional outcome parameters could be found, due to limited availability of these information by the retrospective study design. This underlines the importance of a post-operative interview and clinical examination, to collect detailed and reliable information. Furthermore, the significance of clinical examinations could be increased, by decrease the influence of the examiner. Valentini et al. proposed using electrophysiological measurements to reliably determine the function of peripheral cutaneous nerves [21].

Ghassemi et al. performed a prospective interview with a mixed study group receiving vascular and avascular iliac crest grafts without using CAD/CAM techniques [54]. The correlation between graft volume and postoperative donor-site morbidity was evaluated. Patients with vascularized grafts showed higher graft volumes and were statistically more often exposed to sensible and functional impairment. This confirms the importance of minimal invasive surgery for preventing donor site morbidity.

As CAD/CAM techniques have only been part of the clinical routine for approximately ten years, just one other study (Liu et al.) reports of donor-site morbidity in a study population, in which CAD/CAM was used consistently for DCIA graft harvesting [33]. Instead of determining graft volume by virtual segmentation, Liu et al. collected information about the length of the anterior iliac graft and skin incision to estimate the invasiveness of the surgery. In contrast to our study, no significant correlations with donor-site morbidity could be identified. As the length of the anterior iliac graft was only measured in one dimension by Liu et al., it does not necessarily correlate with the volume of the graft or the invasiveness of the surgery. Thus, the impact of the examined factors may not be detected properly due to methodological drawbacks.

Investigations regarding neurosensory deficits of the pelvis after conventional iliac crest harvesting have been conducted previously [33, 34, 38, 48, 50, 52]. None of the studies found a significant correlation between incision length and neurosensory abnormalities.

The localizations of nerve alterations most frequently stated in literature (iliohypogastric nerve and lateral cutaneous nerve of thigh) are consistent with the findings of this study [34, 48, 52]. Interestingly, strongly varying frequencies of sensory alterations are reported in literature: While some authors report of only a few patients (2%) and short periods of nerve alterations (< 1 month), [38] others report of a great portion of patients (up to 93%) suffering hyp- or anesthesia in the pelvic region [34]. In this study, nerve impairments were found with an incidence of 53%, which is in the middle range of current literature statings. Findings regarding neurosensory deficits may vary due to the heterogeneity of the existent studies: Depending on the applied surgical technique (mono- vs. bicortical, CAD/CAM vs. conventional, microvascular vs. avascular) and study design (chart review vs. questionnaire vs. clinical examination) postoperative deficits may occur with different frequencies and may be detected with varying reliability. Clinical examinations may exhibit more detailed information about the postoperative function of the hip, than mere retrospective chart review. Finally yet importantly, the time of study conduction may influence postoperative findings, as surgical techniques have improved [56].

In general, the small sample size of this study (n = 15) obliges cautious interpretation of the presented results. Anatomical or functional peculiarities of our specific study group may affect statistical analysis, which makes generalizability of our results questionable. Pre- and postoperative CT- or MR-data of the donor sites should be used in future studies to enable a more objective evaluation of the anatomical and functional changes. In fact, it would be useful to compare the results with a control group consisting of conventionally operated patients. Since the advantages of CAD/CAM technology and the associated virtual planning are undeniable, it was considered unethical to conduct a case–control study. Instead, a comparison with existing literature reports on conventionally treated patients was conducted. Nevertheless, advanced investigations with larger study groups may be necessary to reveal the true effect of CAD/CAM driven DCIA graft harvesting on postoperative impairment of the leg.

## Conclusions

Modern CAD/CAM-techniques allow a detailed pre-surgical planning process and aim to improve surgical outcomes. Despite sophisticated preoperative planning options, considerable donor-site morbidity was present in our CAD/CAM-study group. A significant correlation of graft volume with the duration of the use of walking aids and impairment in daily life activities was found, suggesting that surgeons should aim for the smallest possible graft volume in preoperative planning. Additionally, in this study minimally invasive surgical approaches appeared to be superior in terms of neurosensory function, as severe nerve deficits were associated with long skin incisions. The clinical examinations performed showed no statistical correlation of graft volume or skin incision length with ROM or muscle strength. However, preliminary evidence of the association between more minimally invasive surgical approaches and less donor site morbidity after CAD/CAM driven DCIA graft harvesting was obtained. Future studies should conduct clinical trials on larger study groups to determine the impact of surgical invasiveness on postoperative morbidity at the donor site.

## Data Availability

The data presented in this study is available on reasonable request from the corresponding author. The data are not publicly available due to privacy reasons.
